# Specific detection of duck adeno-associated virus using a TaqMan-based real-time PCR assay

**DOI:** 10.3389/fvets.2024.1483990

**Published:** 2024-11-13

**Authors:** Shuyu Chen, YuYi Chen, Mengyan Zhang, Wenyu Zhang, Huanru Fu, Yu Huang, Longfei Cheng, Chunhe Wan

**Affiliations:** ^1^Fujian Key Laboratory for Avian Diseases Control and Prevention, Fujian Academy of Agricultural Sciences, Institute of Animal Husbandry and Veterinary Medicine, Fujian Animal Diseases Control Technology Development Centre, Fuzhou, China; ^2^School of Life Sciences, Fujian Agriculture and Forestry University, Fuzhou, China; ^3^College of Animal Sciences, Fujian Agriculture and Forestry University, Fuzhou, China

**Keywords:** duck adeno-associated virus, DAAV, TaqMan, qPCR, epidemiological surveillance

## Abstract

Duck adeno-associated Virus (DAAV) is a novel pathogen that was recently discovered in ducks. To establish a molecular detection assay for DAAV for further epidemiological investigation and pathogenic mechanism. Here, we designed specific primers and probes according to the sequence characteristics of the newly discovered DAAV and then established a TaqMan real-time PCR method (TaqMan-qPCR) for the detection of DAAV. Our data showed that the established TaqMan-qPCR for detecting DAAV had high sensitivity, with the lowest detection limit of 29.1 copies/μL. No cross reaction was found with duck circovirus (DuCV), H9N2 subtype avian influenza virus (AIV), avian Tembusu virus (ATmV). duck hepatitis A virus 1 and 3 (DHAV-1 and DHAV-3), duck adenovirus A (DAdV-A), duck adenovirus 3 (DAdV-3), or duck enteritis virus (DEV). The repeatability was excellent, with the coefficients of variation of repeated intragroup and intergroup tests ranging from 0.12–0.21% and 0.62–1.42%, respectively. Seventy-eight clinical samples collected from diseased or deceased ducklings were tested. The results showed that the DAAV positive rate was 21.79%, and a triple infection (DAAV+MDPV+GPV) was found. These data provide technical support for further molecular epidemiological surveillance and pathogenic mechanism studies of DAAV infection.

## 1 Introduction

The *Parvoviridae* family consists of linear single-stranded DNA viruses with genomes ranging from ~4 to 6 kb (1). According to the latest classification of The International Committee on Taxonomy of Viruses (ICTV), the viruses of this family are divided into three subfamilies: *Parvovirinae*, which contain viruses that infect vertebrate hosts (2); *Densovirinae*, encompassing viruses that infect arthropods; and *Hamaparvovirinae*, which include viruses from both invertebrates and vertebrates (3). To date, there are 11 genera in *Parvovirinae*, of which adeno-associated virus (AAV) belongs to the genus *Dependoparvovirus* under *Parvovirinae*.

Adeno-associated viruses (AAVs) belong to the Parvoviridae-dependent Parvovirus genus. AAV is a nonenveloped, regular icosahedral, single-stranded DNA with a diameter of 20 to 26 nm and a genome size of 4.7 kb (4). As a replication-defective virus, AAV can replicate and proliferate only under the influence of helper viruses (such as adenovirus and herpes virus) (5). It has two large open reading frames (ORFs) with an inverted terminal repeat (ITR) flanking the open reading frames. The left Rep encodes nonstructural proteins that are important for virus packaging and replication. The right Cap encodes structural proteins (6, 7), which are responsible for the integration, replication, and assembly of viral particles. The infection range of AAV is not limited to dividing cells but also nondividing cells and shows a wide range of tissue tropism *in vivo* (8).

At present, AAV can be divided into three groups, most of which have been detected in mammals (9). Studies have also identified more than 150 viral variants (10, 11). Recently, a duck adeno-associated virus (designated DAAV) was first identified from Muscovy ducks in China (12). Then, DAAV was isolated in Fujian (FJFF001 strain, GenBank accession number: MW286836) in Southeast China using Muscovy duck embryo fibroblasts (MDCEFs). To date, there has been no report on the primers, probes and methods of real-time PCR for the detection of DAAV. Therefore, it is necessary to develop an accurate and efficient DAAV-specific molecular diagnostic platform to help us to better understand the prevalence and transmission of DAAV in waterfowl. This paper describes a novel TaqMan-based real-time polymerase chain reaction method (TaqMan-qPCR) for DAAV, which will help us for further molecular epidemiological surveillance and pathogenic mechanism research of DAAV infection.

## 2 Materials and methods

### 2.1 Viruses

Duck circovirus (DuCV), H9N2 subtype avian influenza virus (AIV), avian Tembusu virus (ATmV), duck hepatitis A virus 1 and 3 (DHAV-1 and DHAV-3), duck adenovirus A (DAdV-A), duck adenovirus 3 (DAdV-3), duck enteritis virus (DEV), goose parvovirus (GPV), Muscovy duck parvovirus (MDPV), and DAAV were stored at Fujian Key Laboratory for Avian Diseases Control and Prevention.

### 2.2 Clinical samples

A total of 78 dead ducklings’ samples (including the spleen, liver, kidney, and intestinal tissue mixture) were collected during 2022–2023 in Southeast China. All dead samples were handled in accordance with the Regulations for the Administration of Affairs Concerning Experimental Animals approved by the State Council of China, which were kindly provided by Professor Bin Jiang. All samples used in this study was obtained by Animal Disease Detection Center, Institute of Animal Husbandry and Veterinary Medicine, Fujian Academy of Agricultural Sciences, which is accredited in accordance with ISO/IEC 17025: 2017 General Requirements for the Competence of Testing and Calibration Laboratories (CNAS-CL01 Accreditation Criteria for the Competence of Testing and Calibration Laboratories) for the competence to undertake the service described in the schedule attached to this certificate (Registration No. CNAS L18448). The 78 samples (including the spleen, liver, kidney, and intestinal tissue mixture) were pooled and regarded as one sample. These samples were homogenized in phosphate-buffered saline (PBS) (20%, w/v). Viral DNA was extracted from duckling sample homogenates via the Magnetic Animal Tissue Genomic DNA Kit.

### 2.3 Primers and probe

According to the sequence characteristics of the novel duck adeno-associated virus (DAAV) gene, specific primer sets (DAAV-TqF and DAAV-TqR) and a probe (DAAV-TqP) for TaqMan-qPCR were designed using Primer Express 3. The primers and probe sequences (Table 1) were subsequently subjected to BLAST^1^ to verify their specificity and synthesized by Sangon Bioengineering Co., Ltd. (Sangon Biotech, Shanghai, China).

**Table 1:** Primers used in this study.

	Primers/probe	Sequence (5′ → 3′)	Location*^1^	Amplicon size (bp)
cPCR	DAAV-F	GAGCACGACAAGGCGTACGA	250–269	617
	DAAV-R	CAGTGGAATCGGTTAAAGTCA	846–866	
TaqMan-qPCR	DAAV-TqF	GTAACCTCGGTAAAGCGGTATT	365–386	100
	DAAV-TqR	TTTCGACGTTCGTTGGTAGG	445–464	
	DAAV-TqP*^2^	TTGGCTTGGCTGAAGACGGAAAGA	416–439	-

*^1^The location was calculated according to the FJFF001 strain (GenBank accession number: MW286836). *^2^The fluorescent reporter dyes FAM and DBQ1 were labeled at the5’-end and 3′-end of DAAV-TqP.

### 2.4 Standard plasmids

The EasyPure Viral DNA/RNA Kit (TransGen Biotech, Beijing, China) was used to extract the nucleic acid DNA of the DAAV-FJ strain (GenBank accession number: MW380871), and then the Cap gene fragments were amplified using DAAV-F and DAAV-R by conventional PCR (cPCR) technology (Table 1). After PCR, the PCR products (approximately 617 bp) were identified by electrophoresis on a 1.0% agarose gel (TransGen Biotech, Beijing, China) and then cloned and inserted into the pEASY-T1 vector according to the instructions of the pEASY-T1 Simple Cloning Kit (TransGen Biotech, Beijing, China). The obtained recombinant plasmids were sent to Sangon Bioengineering (Shanghai, China) Co., Ltd. for sequencing. The sequencing results were verified by BLAST analysis in NCBI. The positive recombinant plasmids (T-DAAV-Tq) were used as the positive standard of TaqMan-qPCR and quantified using an ND-2000c spectrophotometer (NanoDrop2000, Wilmington, United States). The copy number was calculated to be 2.91 × 10^10^ copies/μL according to a previous study (13), using the formula: Amount (copies/μL) = [DNA concentration (g/μL)/(plasmid length in bp × 660)] × 6.02 × 10^23^. Ten-fold dilutions of T-DAAV-Tq, ranging from 2.91× 10^9^–2.91 × 10^0^ copies/μL, were prepared using TE buffer (10 mmol/L Tris–HCl, 1 mmol/L EDTA). All aliquots of each dilution were stored at −80°C until use.

### 2.5 TaqMan-qPCR reaction

TaqMan-qPCR was prepared according to the instructions of the Premix Ex Taq™ (Probe qPCR) kit (TaKaRa Biotechnology, Dalian, China), and the total reaction volume was 25 μL. To determine the optimal primer amount of the reaction, 0.1–1.0 μL (10 μmol/L) was used for the reaction, and different annealing temperatures (ranging from 54 to 62°C) were set for the reaction to determine the optimal annealing temperature. Tenfold serial dilutions of T-DAAV-Tq (ranging from 2.91× 10^6^–2.91 × 10^1^ copies/μL), were used to generate the standard curve under the optimal reaction conditions using LightCycler 96 Instrument (Mannheim, Germany). The plasmid copy number logarithm was plotted against the corresponding Ct values (cycle number threshold), and the standard curve was obtained.

### 2.6 Sensitivity, specificity, and repeatability

To evaluate the sensitivity, serial dilutions of T-DAAV-Tq (ranging from 2.91 × 10^3^–2.91 × 10^0^ copies/μL) were used as the template, and optimized TaqMan-qPCR was used for detection. To evaluate the specificity, nucleic acid DNA was extracted from DuCV, AIV, ATmV, DHAV-1, DHAV-3, DAdV-A, DAdV-3, DEV, GPV and MDPV. To evaluate the reproducibility, DAAV-positive standard samples with three different concentrations (2.91 × 10^6^ copies/μL, 2.91 × 10^4^ copies/μL, 2.91 × 10^2^ copies/μL) were used as templates. Three replicates were performed for each concentration of plasmid, to determine the coefficient of variation (CV). The intragroup and intergroup with the coefficient of variation (CVs) for Ct values were calculated.

### 2.7 Clinical testing

A total of 78 samples were teste using the established TaqMan-qPCR method. The weight of each sample was 25 mg and was homogenized with sterile PBS in a mortar. Then, the suspensions were collected after centrifugation at 4,000 rpm at 4°C for 15 min. Nucleic acid was extracted from the supernatant using the Magnetic Animal Tissue Genomic DNA Kit (Tiangen Biotech, Beijing, China) according to the manufacturer’s instructions. Because GPV and MDPV also belong to the genus *Dependoparvovirus* under the *Parvoviridae* family, the GPV and MDPV were also detected in the above samples by the real-time PCR previously reported by us (14, 15), which will help us to learn about the co-infection situation of duck-origin related diseases under the *Parvoviridae* family.

## 3 Results

### 3.1 TaqMan-qPCR optimization

The optimal reaction system of 25 μL was optimized by the established TaqMan-qPCR assay as follows: Premix Ex Taq™ (Probe qPCR) master mix 12.5 μL, 0.4 μL each of DAAV-TqF and DAAV-TqR primers (10 μmol/L), and probe (DAAV-TqP, 5 μmol/L), template (1 μL), and sterilized ddH_2_O to a final volume of 25 μL. The optimized reaction conditions were as follows: 95°C for 120 s and 40 cycles of 95°C for 10 s and 60°C for 30 s.

### 3.2 Standard curve

**Figure 1:**
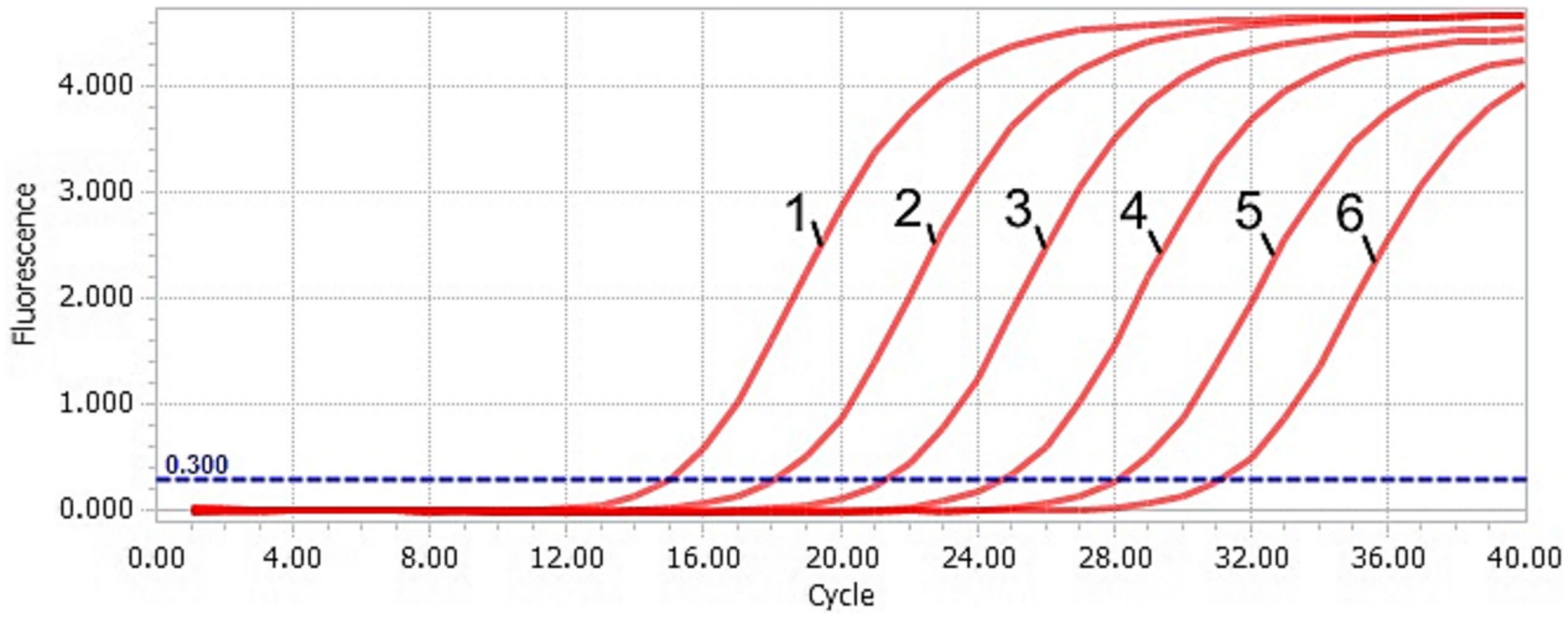
Amplification curve of the TaqMan-qPCR assay. 1–6: plasmid concentration ranging from 2.91 × 10^6^–2.91 × 10^1^ copies/μL.

**Figure 2:**
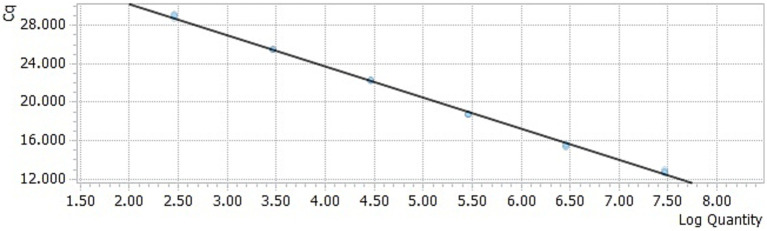
Standard curve of the TaqMan-qPCR assay.

The DAAV real-time PCR standard curve was obtained by taking the common logarithm (lgC) of copy number in each concentration standard template as the horizontal coordinate and cycle threshold (Ct value) as the ordinate (Figures 1, 2). The slope of the standard curve was −3.2634, the Y-intercept was 36.787, the R^2^ was 0.9987, and the amplification efficiency was 99%, which indicated that the standard curve of the TaqMan-qPCR has a good linear relationship.

### 3.3 Sensitivity, specificity, and repeatability

**Figure 3:**
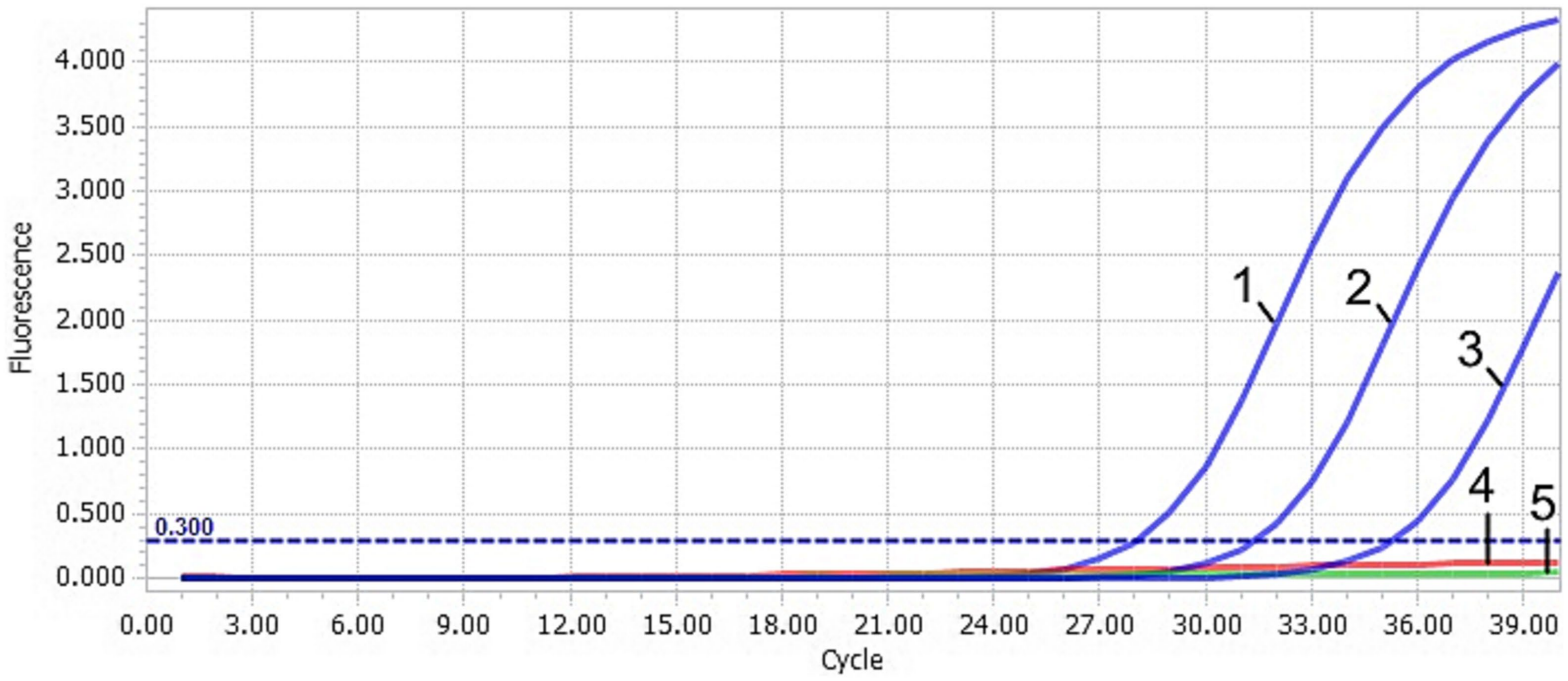
Sensitivity of the TaqMan-qPCR assay. 1–4: plasmid concentration ranging from 2.91 × 10^3^–2.91 × 10^0^ copies/μL. 5: negative control (ddH_2_O).

**Figure 4:**
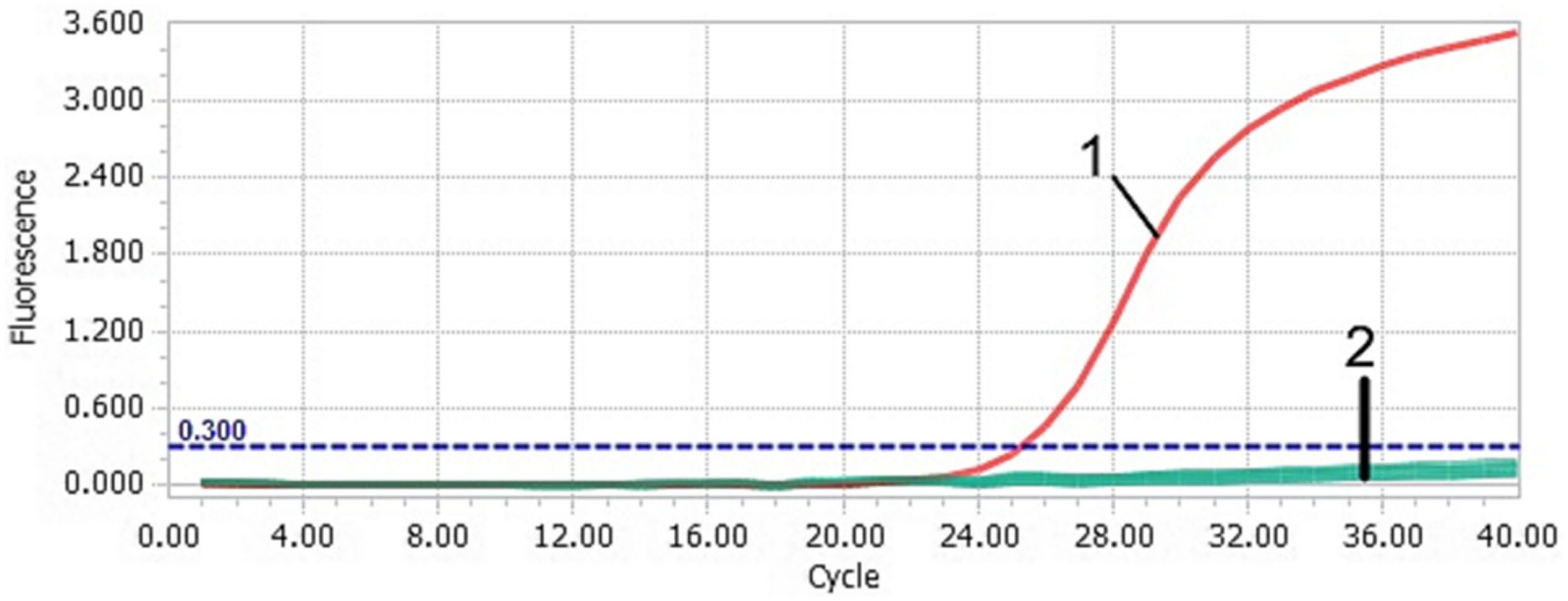
Specificity of the TaqMan-qPCR assay. 1: DAAV; 2: DuCV, AIV, ATmV, DHAV-1, DHAV-3, DAdV-A, DAdV-3, DEV, GPV, MDPV and ddH_2_O. These controls did not yield a positive fluorescent signal. It cannot be effectively distinguished by the naked eye.

After TaqMan-qPCR amplification, the minimum detection limit of the assay was 2.91 × 10^1^ copies/μL (29.1 copies/μL) (Figure 3). Using the viral DNA and cDNA as template (DuCV, AIV, ATmV, DHAV-1, DHAV-3, DAdV-A, DAdV-3, DEV, GPV and MDPV), the data showed only DAAV with a positive signal, while other templates followed with no response signal (Figure 4). Similar data were observed after three independent reactions were repeated. The results showed that TaqMan-qPCR was highly specific. The intragroup coefficient of variation of the assay was 0.12–0.21%, and the intergroup coefficient of variation was 0.62–1.42%. The results are shown in Table 2, demonstrating the good repeatability of the assay.

**Table 2:** Coefficient of variation of the TaqMan-qPCR assay.

Serial number	Copies	Intra-assay CVs			Inter-assay CVs		
		Ct	Standard deviation	CV (%)	Ct	Standard deviation	CV (%)
1	2.91 × 10^6^	15.46	0.12	0.76	15.52	0.10	0.62
2	2.91 × 10^4^	22.24	0.09	0.41	22.31	0.15	0.67
3	2.91 × 10^2^	28.90	0.21	0.72	28.99	0.42	1.42

### 3.4 Clinical samples evaluation

#### 3.4.1 Duck adeno-associated virus evaluation

The 78 collected clinical samples were tested using the established TaqMan-qPCR assay (Table 3). For DAAV, 17 samples tested positive, with a positive ratio of 21.79% (17/78). Moreover, the TaqMan-qPCR-positive samples were also verified by cPCR method (described at Section2.4, using DAAV-F and DAAV-R), 14 of 17 were tested with cPCR-positive. To confirm the presence of DAAV in cPCR-positive samples, the expected product sizes were cloned (described at Section2.4). For each PCR product, three colonies were selected for Sanger sequencing in both directions at Sangon (Shanghai, China). In terms of sequence identity, the identified DAAV-cPCR-positive viruses showed nucleotide of 100% with FJFF001 (GenBank No. MW286836).

**Table 3:** Detection results for the clinical samples.

Species	Sample number	DAAV		MDPV		GPV		DAAV+MDPV		DAAV+GPV		DAAV+MDPV+GPV	
		N*	R*	N*	R*	N*	R*	N*	R*	N*	R*	N*	R*
Muscovy duck	34	7	18.92	13	35.14	1	2.86	6	17.65	1	2.86	1	2.86
Cherry Valley Duck	27	5	18.52	0	0	8	29.63	0	0	4	14.81	0	0
shelduck	17	5	29.41	0	0	7	17.07	0	0	2	11.76	0	0
Total	78	17	21.79	13	16.67	16	20.51	6	7.69	7	8.97	1	1.28

N* means positive number, R* means positive ratio (%).

#### 3.4.2 Muscovy duck parvovirus and GPV coinfection evaluation

For MDPV, 13 samples tested positive, with a positive ratio of 16.67% (13/78). For GPV, 16 samples tested positive, with a positive ratio of 20.51% (16/78). Moreover, the DAAV and MDPV coinfection positive ratio was 7.69% (6/78), the DAAV and GPV coinfection positive ratio was 8.97% (7/78), and the DAAV, MDPV and GPV triple infection rate was 1.28% (1/78), with the copy number of 8.75 × 10^3^ copies/μL(for DAAV), 3.92 × 10^4^ copies/μL(for MDPV), and 7.09 × 10^3^copies/μL(for GPV), respectively.

## 4 Discussion

In recent years, the emergence of new duck viral diseases has caused enormous losses to waterfowl breeding in China. Therefore, the exploration of unknown duck-origin viruses is conducive to the prevention and control of waterfowl diseases. Adeno-associated virus (AAV), a kind of single-stranded DNA-defective virus, is the smallest type of animal virus due to its simple genetic structure and small size (16, 17). AAV is widely used in the construction of gene expression vectors and gene therapy because of its good safety, wide host range and low immunogenicity (18, 19). A novel duck adeno-associated virus (DAAV) was identified during the virological investigation of waterfowl disease in the Muscovy ducks. Due to limited information, the specific pathogenicity of DAAV remains to be fully understood, and accurate measurement of viral loads in different tissues of DAAV-infected ducks could allow researchers to more fully understand the relationship between this virus and the onset or progression of the disease.

Fluorescence PCR is widely used in the diagnosis of clinical diseases due to its high sensitivity and good specificity (20). Real-time fluorescence PCR technology based on TaqMan fluorescence-labeled probes is the most widely used in clinical diagnosis in China (14, 15, 21–23). The TaqMan fluorescent probe is labeled with a fluorescent reporter group at the 5′ end and a quenching agent at the 3′ end. Based on this principle, we analyzed the DAAV Cap gene and established a TaqMan-based real-time PCR assay for the detection of DAAV.

In the present study, the establishment and evaluation of the TaqMan-qPCR for fast, sensitive and accurate detection of DAAV was used. Our data were analytically specific and sensitive and presented excellent intra- and inter-assay CVs (both less than 1.50%), demonstrating that the established TaqMan-qPCR assay is a reliable and reproducible platform. For clinical evaluation, the positive rate of DAAV, MDPV and GPV were 21.79, 16.67 and 20.51%, respectively. The coinfection rates of DAAV+MDPV and DAAV+GPV was 7.69 and 8.97%, respectively; moreover, we also found a triple infection (DAAV+MDPV+GPV) in Muscovy duck. The role of DAAV and its coinfection with GPV and MDPV needs further study.

## 5 Conclusion

In conclusion, our study provides a DAAV detection platform using the TaqMan-qPCR assay with good sensitivity, specificity, and repeatability. This platform will help us further molecular epidemiological surveillance and pathogenesis studies.

## Data Availability

The original contributions presented in the study are included in the article/supplementary material, further inquiries can be directed to the corresponding author/s.

## Footnotes

^1^ https://blast.ncbi.nlm.nih.gov/Blast.cgi
