# Corrigendum: Specific detection of duck adeno-associated virus using a TaqMan-based real-time PCR assay

**DOI:** 10.3389/fvets.2024.1539901

**Published:** 2025-01-13

**Authors:** Shuyu Chen, YuYi Chen, Mengyan Zhang, Wenyu Zhang, Huanru Fu, Yu Huang, Longfei Cheng, Chunhe Wan

**Affiliations:** ^1^Fujian Key Laboratory for Avian Diseases Control and Prevention, Fujian Academy of Agricultural Sciences, Institute of Animal Husbandry and Veterinary Medicine, Fujian Animal Diseases Control Technology Development Centre, Fuzhou, China; ^2^School of Life Sciences, Fujian Agriculture and Forestry University, Fuzhou, China; ^3^College of Animal Sciences, Fujian Agriculture and Forestry University, Fuzhou, China

**Keywords:** duck adeno-associated virus, DAAV, TaqMan, qPCR, epidemiological surveillance

In the published article, there was an error in Table 2 as published. The Ct value of Inter-assay CVs should be changed as,

15.52, instead of 15.46;

22.31, instead of 22.24;

28.99, instead of 28.90.

The corrected Table 2 and its caption appear below.

**Table 2:** Coefficient of variation of the TaqMan-qPCR assay.

**Serial number**	**Copies**	**Intra-assay CVs**			**Inter-assay CVs**		
		**Ct**	**Standard deviation**	**CV (%)**	**Ct**	**Standard deviation**	**CV (%)**
1	2.91 × 10^6^	15.46	0.12	0.76	15.52	0.10	0.62
2	2.91 × 10^4^	22.24	0.09	0.41	22.31	0.15	0.67
3	2.91 × 10^2^	28.90	0.21	0.72	28.99	0.42	1.42

The authors apologize for this error and state that this does not change the scientific conclusions of the article in any way. The original article has been updated.

